# The Emerging Role of Small Extracellular Vesicles in Inflammatory Airway Diseases

**DOI:** 10.3390/diagnostics11020222

**Published:** 2021-02-02

**Authors:** Katarzyna Piszczatowska, Katarzyna Czerwaty, Anna M. Cyran, Mathias Fiedler, Nils Ludwig, Jacek Brzost, Mirosław J. Szczepański

**Affiliations:** 1 Department of Biochemistry, Medical University of Warsaw, 02-091 Warsaw, Poland; kpiszczatowska@wum.edu.pl; 2 Department of Otolaryngology, The Medical Centre of Postgraduate Education, 01-813 Warsaw, Poland; katarzynaczerwaty@gmail.com; 3 Department of Pathology and Laboratory Medicine, Brown University, Providence, RI 02912, USA; anna_cyran@brown.edu; 4 Department of Oral and Maxillofacial Surgery, University Hospital Regensburg, 93053 Regensburg, Germany; mathias1.fiedler@stud.uni-regensburg.de (M.F.); nils.ludwig@klinik.uni-regensburg.de (N.L.); 5 The Children’s Memorial Health Institute, 04-730 Warsaw, Poland; jbrzost@vp.pl

**Keywords:** small extracellular vesicles, exosomes, inflammatory airway diseases, chronic rhinosinusitis, otitis media, lung diseases, bronchial diseases, inflammation

## Abstract

Extracellular vesicles (EVs) are produced and released by all cells and are present in all body fluids. They exist in a variety of sizes, however, small extracellular vesicles (sEVs), the EV subset with a size range from 30 to 150 nm, are of current interest. By transporting a complex cargo that includes genetic material, proteins, lipids, and signaling molecules, sEVs can alter the state of recipient cells. The role of sEVs in mediating inflammatory processes and responses of the immune system is well-documented, and adds another layer of complexity to our understanding of frequent diseases, including chronic rhinosinusitis (CRS), asthma, chronic obstructive pulmonary disease (COPD), and upper airway infections. In these diseases, two aspects of sEV biology are of particular interest: (1) sEVs might be involved in the etiopathogenesis of inflammatory airway diseases, and might emerge as attractive therapeutic targets, and (2) sEVs might be of diagnostic or prognostic relevance. The purpose of this review is to outline the biological functions of sEVs and their capacity to both augment and attenuate inflammation and immune response in the context of pathogen invasion, CRS, asthma, and COPD.

## 1. Introduction

**Figure 1:**
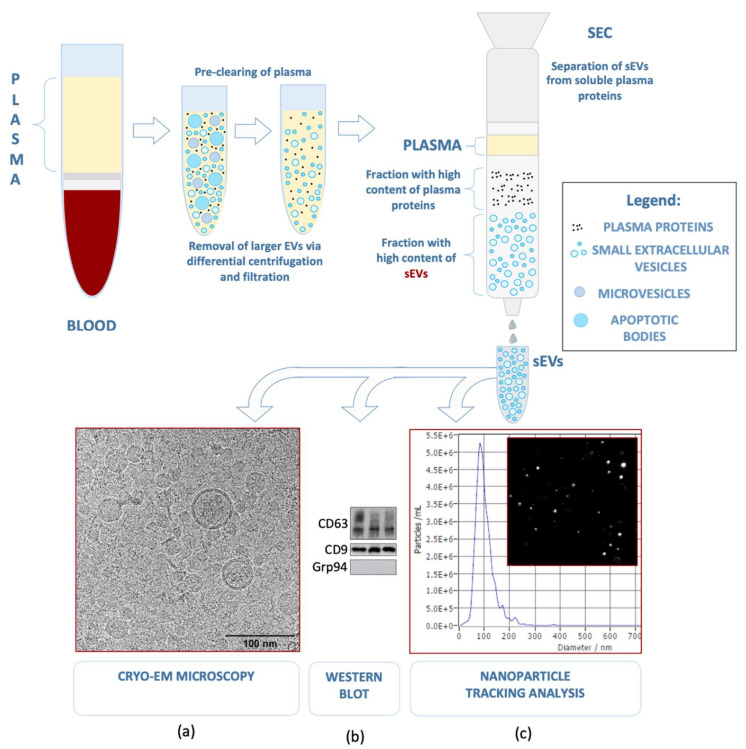
Small extracellular vesicles present in human plasma may be separated using pre-clearing with differential centrifugation and a 200 nm filter (not shown), followed by size exclusion chromatography. The isolated sEVs can be characterized according to the guidelines of the International Society for Extracellular Vesicles [4] with the use of (**a**) Cryo-EM microscopy (52,000×) to estimate their size and morphology, (**b**) western blot for two positive (CD63 and CD9) and one negative (Grp94) sEV marker, and (**c**) nanoparticle tracking analysis (NTA), which allows the evaluation of vesicle size (average diameter = 90.9 nm) and concentration (1.3 × 10^11^ particles/mL) [9] (modified).

Extracellular vesicles (EVs) are produced and released by all cells and are present in all body fluids. They exist in a variety of sizes, but of particular interest are small extracellular vesicles (sEVs), which range in size from 30 to 150 nm [1,2,3]. They originate from the endocytic compartment of the producer cell, and, because of their endosomal origin, sEVs are distinct from larger EVs, such as microvesicles (MVs), which are formed by “pinching off” the cellular membrane or from apoptotic bodies. Despite extensive research on EVs, their nomenclature is not fully established, leading to possible overlaps among various EV subtypes. According to the newest guidelines of the International Society for Extracellular Vesicles [4], we decided to use the term sEVs in this review for vesicles that are often also referred to as exosomes in the literature. A growing body of evidence indicates that sEVs play a major role in intercellular communication in physiological as well as in pathological conditions [5,6,7]. While the initially suspected role of sEVs was in eliminating cellular waste products [8], the growing interest in sEVs has led to an active international research community as well as improved isolation methods [9] that continuously broaden our understanding of sEV biogenesis, structure, and functions (Figure 1).

sEVs are uniquely positioned to mediate immune response and inflammatory reactions [10]. In recent years, research interest in sEVs has surged; sEVs have been linked to a number of human pathologies i.e., chronic rhinosinusitis, asthma, and airway infections, which are multifactorial in etiology, but unfailingly associated with excessive stimulation of the immune system. sEVs are present in many types of body fluids, including blood [11,12], urine [13], saliva [14], bronchioalveolar lavage fluid (BALF) [15], lymph [16], and nasal lavage fluid [17]. Therefore, sEVs are considered an attractive opportunity for non-invasive diagnostics with regards to their potential use as a liquid biopsy. In addition, sEVs might be a promising approach to monitor disease progression or response to therapy, as shown for malignant diseases [18]. First attempts have been made to harness the properties of sEVs and use them for drug delivery [19], and thus utilize them as therapeutic vesicles [20]. EV-based therapeutics are currently being developed to treat cancer, as well as inflammatory and autoimmune diseases [21].

In this article, we discuss the contribution of sEVs to inflammatory conditions of the respiratory tract. Our focus will be chronic rhinosinusitis with nasal polyps (CRSwNP) or without nasal polyps (CRSsNP), acute upper airway infections, asthma, and chronic obstructive pulmonary disease (COPD). We will present the role of sEVs in the group of airway diseases that are associated with a strong inflammatory background. We decided to exclude cancers of the respiratory system because of their wide etiologic spectrum that needs to be broadly and particularly addressed. We also address the multifaceted role of sEVs in infection and their interplay with pathogens, to which the airway epithelium is invariably exposed. Lastly, we outline the diagnostic and therapeutic possibilities.

## 2. Small Extracellular Vesicles—Biogenesis, Cargo Components, and Functions

### 2.1. Biogenesis

The biogenesis of sEVs begins by directing cargos intended for secretion to the early endosomes (EEs). EEs accumulate intraluminal vesicles and later convert to multivesicular bodies (MVBs) [5,22]. Upon invagination of the endosomal membrane, a portion of cytoplasm is engulfed within the newly formed vesicle. Most MVBs later fuse with lysosomes, which ensures the degradation of their content by hydrolases. However, vesicles harbouring CD63, LAMP1, LAMP2, and MHCII can avoid degradation and fuse with the plasma membrane, releasing sEVs into the intercellular space [1,5].

Four endosomal sorting complexes required for transport (ESCRT) play a key role in both cargo creation and vesicle separation [23]. In the first step, ESCRT-0 and ESCRT-I direct cargos to the assembly site. Then, ESCRT-II and -III facilitate sEV budding and fission; sEVs may also be formed in the absence of ESCRT. Several mechanisms have been described, and one of them involves the formation of transmembrane protein clusters composed of tetraspanins and other proteins at the sites of MVBs, which then invaginate to form a vesicle. These mechanisms are distinct but overlapping, and each cell likely features a population of EVs from different origins [24]. TSG101 (tumor susceptibility gene 101 protein), ALIX, and VPS4 (vacuolar protein sorting-associated protein 4) are proteins carried by EVs that can be used to determine the origin from MVBs. Syndecan-1 and syntenin-1 were demonstrated to interact with ALIX and ESCRT-I and -III, and might be involved in the formation of sEVs [25]. The trafficking and secretion is mediated by small GTPases from the Rab family, for instance Rab27a and Rab27b [26]. Another crucial process is the merging of MVBs with the plasma membrane, which is mediated by SNARE complexes (soluble *N*-ethylmaleimide sensitive fusion attachment protein receptor) [25].

### 2.2. Cargo Components

**Figure 2:**
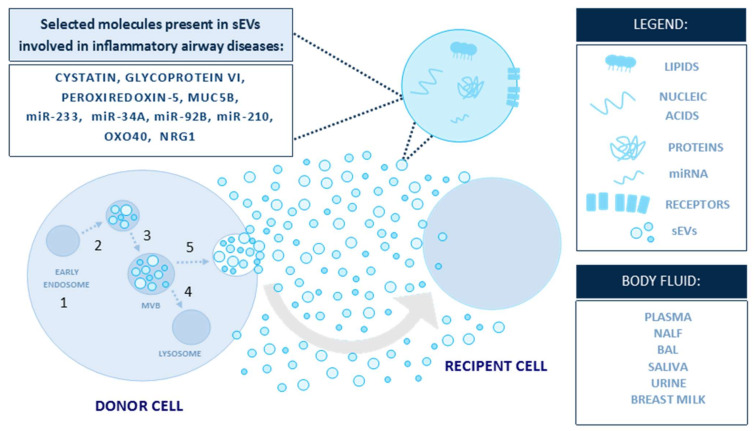
sEVs are carriers of a complex biologically active cargo [27] (modified). The figure presents selected molecules carried within sEVs that are involved in inflammatory airway diseases. The biogenesis of sEVs begins when cargos for secretion are located in early endosomes, (1) which accumulate intraluminal vesicles (2) and later convert to multivesicular bodies (MVBs) (3). MVBs might fuse with lysosomes and degrade (4) or fuse with the plasma membrane, (5) releasing sEVs into the intercellular space.

The cargo composition of sEVs highly depends on the cell of origin, as well as on the status of the secreting cell [27,28]. A comprehensive analysis of nucleic acids enclosed in sEVs has shown a distinct repertoire of extracellular RNA [29], as well as the presence of DNA associated with sEVs [4,30]. Furthermore, proteomic analyses have revealed members of various cellular pathways, including cytoskeletal components, annexins, signal transducers, metabolic enzymes, and chaperone proteins; sEVs originating from antigen-presenting cells carry major histocompatibility complex (MHC) molecules and costimulatory molecules CD86 and CD54 [31]. Certain protein families are particularly abundant. The most notable examples are tetraspanins, a group of transmembrane proteins with a role in cell aggregation and motility [32]. Tetraspanins may act as molecular traps, binding to a variety of proteins and directing them to sEVs. Some family members, such as CD81, CD63, and CD9, have been proposed as sEV markers [5] (Figure 2). Nevertheless, the molecular composition of sEVs is much narrower than the repertoire of the parent cell. Although these patterns are only beginning to emerge, current observations suggest the existence of an elaborate mechanism governing the inclusion of molecules to sEVs. Due to the biological structure of sEVs, their cargo components can either be associated with the sEV membrane or they can be enclosed in the vesicle lumen [24] (Figure 2, Table 1). To distinguish the cellular origins of sEVs or the type of disease, several specific abbreviations were established in the literature. For instance: tumor-derived sEVs: TEX [33], nasal mucus-derived sEVs: rhinosomes [34], and dendritic cell-derived sEVs: DEX [35].

### 2.3. Functions

sEVs were first described as vesicles secreted upon fusion with the plasma membrane by maturing reticulocytes [36]. This observation has sparked the idea that sEVs present an alternative route to eliminate molecules no longer needed for cellular homeostasis or ones resistant to lysosomal degradation. Raposo et al. revealed that sEVs originating from B-cells harbor a functional MHCII complex, and are capable of inducing an antigen-specific T cell response [37]. These findings paved the path for further discoveries of sEV functions in antigen presentation, immunosuppression, mediation of inflammation, and viral infection [24]. However, the functions of sEVs appear to be even more complex, and sEVs are now considered to play a crucial role in cell-to-cell communication [6]. They are capable of reprogramming recipient cells by transporting mRNAs and miRNAs, which are able to trigger the translation of specific proteins [1]. Their role in transmitting chemokines, cytokines, and other signaling molecules is of particular interest, as the complexity of the sEV cargo composition has the capacity to induce effects on recipient cells. Several biological functions of sEV cargo components have been described in the literature, including angiogenic/anti-angiogenic effects, tissue regeneration, immune cell activation, or immunosuppression, as well as metastasis and cancer progression; sEVs derived from human mesenchymal stroma cells might have promising therapeutic potential in allergic airway inflammation. Inhibition of ILC2 (innate lymphoid cells), infiltration of inflammatory cells, decreased production of mucus in the lung, and reduced Th2 cytokine levels were associated with human mesenchymal stroma cell-derived sEVs; miR-146a transported by this EV type could be responsible for these effects [38]. The biological functions of sEVs are summarized in Table 1.

## 3. Role of sEVs in Inflammatory Airway Diseases

Inflammatory airway diseases are complex with regards to their heterogeneous etiologies. The literature about sEVs in inflammatory airway diseases addresses different aspects of sEV biology, however, the diagnostic/prognostic values of sEVs and their role in etiopathogenesis are the most frequently investigated topics. In the following sections, we will focus on different inflammatory airway diseases and present the available sEV-based literature, with special emphasis on diagnostic/prognostic or etiopathologic aspects of sEVs. Our main focus will be the role of sEVs in mediating inflammation and immune responses.

Inflammation is triggered by antigen presentation, as well as stimulation by cytokines, chemokines, and other signaling molecules. The complex inflammatory cascade consists of several steps that have been shown to be influenced by sEVs, suggesting that sEVs can play a pro- and anti-inflammatory role. The cargo composition of sEVs is considered to reflect the state of the parent cell [57,58] and, therefore, depending on the cell of origin, they may carry a cocktail of signaling molecules and other inflammatory substrates. Examples include the trafficking of enzymes for leukotriene biosynthesis [59] and Hsp70, which induces the production of tumor necrosis factor-α (TNF-α), interleukin 13 (IL-13), and interferon-γ (IFN-γ) in target cells [60]; sEVs can also engage immune cells. Dendritic cells exchange miRNAs, which are encapsulated in sEVs and alter gene expression and direct immune response in accordance with the specific miRNA sequence carried [61]. Moreover, antigen-presenting cell (APC)-derived sEVs can directly stimulate naïve T cells. MHC complexes on sEVs are recognized by CD8+ lymphocytes, despite the absence of APC, and invoke an immunogenic response in the presence of co-stimulators [62].

### 3.1. Upper Airways

#### 3.1.1. sEVs and Chronic Rhinosinusitis (CRS)

Chronic rhinosinusitis is a heterogeneous disease involving inflammation of the sinonasal mucosal lining. It is a prevalent problem, adversely affecting the quality of life of 5–12% of the global population. The traditional phenotypic classification into CRS with and without nasal polyps failed to account for the diverse molecular pathomechanisms underlying the disease. The publications about CRS of the last 10 years have led to a paradigm shift in the understanding of this disease. It is now considered as a disease resulting from a maladjusted interplay between environmental cues (pathogen invasion, microbiome, and permeability of mucosal lining) and the immune system. The European Position Paper on Rhinosinusitis and Nasal Polyps 2020 [63] turns away from the phenotypic classification and focuses on the pathophysiology of the disease instead. Based on the endotype, CRS is now divided into primary and secondary. It is further defined by anatomic localization and endotype dominance, classified either into type 2 (associated with more severe manifestations and resistance to therapy) or non-type. Likewise, secondary CRS is characterized as localized or diffuse, and further defined by endotype dominance [63]. This approach focuses on upstream regulators rather than manifestations, paving the way for personalized, etiology-driven therapies.

The ability to reproducibly and non-invasively obtain and analyze sEVs from nasal fluids could offer the possibility of defining CRS endotypes, aligning them with clinical outcomes, and introducing them into a routine diagnostic workup. A proteomic analysis of such samples from patients with CRS showed 123 differentially expressed proteins, pointing to over 40 dysregulated signaling pathways. Significant differences in sEV proteome were found between CRS with polyps (CRSwNP) and the control group. Among the most promising molecular markers of CRSwNP were cystatin, glycoprotein VI, and peroxiredoxin-5 [39]. In another study, high levels of epithelial protease inhibitors cystatin-1 and -2 were found in sEVs. Based on this finding, cystatin-2 was proposed as a marker for CRS, capable of predicting the disease phenotype [40]; sEVs may also contribute to the formation of polyps due to the upregulation of pappalysin and serpins [41,42].

One of the hallmarks of the CRS phenotypes is the imbalance between Th1 and Th2 cells. While CRSwNP relies on Th2 cells, CRSsNP is dominated by the Th1 response; sEVs can shift this equilibrium by promoting the differentiation of Th2 and suppressing Th1 lymphocytes [43]. Ickrath et al. have shown that tissue samples from patients with CRSwNP feature higher levels of CD8+ than CD4+ T cells. Their study also suggests the possibility of a local regulation mechanism within the polyp microenvironment [64]. Interestingly, studies have shown that sEVs can have an effect on the profile of T cells. Stimulation of resting CD3+ T cells with IL-2 and sEVs from their activated counterparts shifted the T cell profile to CD8+ and changed the cytokine profile [65]. Studies in mouse models demonstrated that mast cell-derived sEVs may impact B and T cell functions, ultimately contributing to inflammation [66]. It was shown that sEVs are transported through the lymphatic system from the periphery to the lymph node, and that B cells, together with macrophages, are key players in sEV uptake [16].

#### 3.1.2. sEVs and Airway Epithelium

The airway epithelium is the first line of defense against pathogens, and consists of several different barriers to prevent pathogen invasion. Its antimicrobial properties include secretion of lysozyme, lactoferrin, hydrogen peroxide, nitric oxide, and mucins. Epithelial cells express toll-like receptors (TLRs), capable of recognizing pathogen-associated molecular patterns (PAMPs) and activating an immune cell response [67]. Recent observations showed that sEVs can modulate the innate immune response in the airway. Bacterial lipopolysaccharides (LPSs) recognized by TLR4 increased the production of sEVs by epithelial cells, which carry nitric oxide synthase [68]. In CRSsNP, the expression of TLR2 and TLR4 correlates with neutrophil abundance [69], and it was demonstrated that sEVs correspond to the expression of TLR receptors in airway epithelium [70].

Nasal polyps are benign outgrowths of sinonasal mucosa, characterized by increased epithelial cell proliferation, interstitial edema, and increased angiogenesis. Interestingly, sEVs isolated from the epithelium of CRSwNP patients contain proteins participating in proliferative pathways and enzymes known for their role in angiogenesis, suggesting that sEVs are involved in inflammatory tissue remodeling [44,46]. CRSwNP-derived sEVs contain high levels of permeability glycoprotein (P-gp), which regulates cytokine secretion [34].

The structures of the middle ear, which connect to the upper respiratory tract and nasal cavity via the Eustachian tube, are also covered with a respiratory-type epithelium. The middle ear epithelium (MEE) plays an important role in the development of middle ear otitis (OME), and is composed of ciliated cells, secretory cells, non-secretory cells, and basal cells. Secretory cells are responsible for the production of mucins and various anti-microbial proteins, such as lactotransferrin, lysozyme, defensins, and surfactants [71,72]. Val et al. were the first to isolate sEVs from the middle ear fluid samples of 16 pediatric patients. Not unexpectedly, the proteomic analysis showed an enrichment in neutrophil markers and molecules associated with innate immunity, such as immunoglobulins, MUC5B, and heat-shock proteins; also related to neutrophil stimulation were 29 enriched miRNA sequences unique to the middle ear samples, including the most abundant miR-223 [45]. On the cellular level, it was shown that human middle ear epithelial cells treated with Haemophilus influenzae lysate secrete sEVs containing heterogeneous nuclear ribonucleoproteins, such as hnRNP A2B1 and hnRNP Q, and also miRNAs that might be involved in immunity regulation [47].

It is also suspected that sEVs play a role in the pathogenesis of chronic otitis media with cholestatoma. In middle ear cholestatoma patients, sEVs derived from keratinocytes might induce osteoclast differentiation. This was observed after the addition to fibroblasts co-cultured with osteoclast precursor cells. Downregulated miR-17 enclosed in sEVs, which regulates Tnfsf11 expression in fibroblasts, appears to be responsible for this effect [48]. Another in vitro study showed that human cholesteatoma perimatrix fibroblast-derived sEVs promote angiogenesis through downregulation of miR-106b-5p in sEVs, leading to the overexpression of Angiopoietin-2 in human umbilical vein endothelial cells (HUVECs). Furthermore, sEVs contributed to enhanced tube formation and cell migration [49]. These findings posit sEVs as important factors in the pathogenesis and progression of OME and chronic otitis media with cholesteatoma.

#### 3.1.3. sEVs and Bacteria in Upper-Airway Inflammation

There is a close cooperation between the host’s immunity and the microbiome. Chronic inflammatory processes, such as CRS, are often associated with decreased microorganism diversity and an imbalance within the microbiome, which likely contributes to the perpetual inflammatory signaling. This notion is confirmed by observations that potentially pathogenic bacterial species may be present, albeit in low proportions, in healthy patients [73,74]. At the same time, sEVs secreted by microbiological flora shape the host’s immune response, and can be a causative factor in inflammatory conditions [75]. Bacteria-derived sEVs contain various molecules, including proteins, nucleic acids, lipids, and glycans. It is likely that these sEVs impact different cytotoxic and immunomodulatory mechanisms to ultimately facilitate the survival of the pathogen. Another important fact is that bacteria-derived sEVs transport LPS that is known for its immunomodulatory functions [76]. Bacterial EVs were shown to interact with various cells, including dendritic cells, macrophages, and neutrophils; sEV-associated PAMPs allow binding to receptors present on the surface of immune cells, and activate immune response pathways that result in pro-inflammatory cytokine production. Additionally, it is suggested that sEVs may convey antigens to stimulate the immune response [77,78]. Participation of bacterial-derived sEVs in the regulation of gene expression is also suspected. For example, *Pseudomonas aeruginosa*-derived sEVs were able to suppress the expression of the group of MHC proteins in lung macrophages [79]. On the other hand, some data indicate a positive and non-pathogenic role of bacteria-derived sEVs to the functionality of the healthy microbiome [80]. Metagenomic analysis of bacteria-derived sEVs isolated from the nasal lavage fluid of CRS patients also revealed their high abundance with decreased diversity. Several differences between CRS and non-CRS patients were discovered, since some bacterial groups were decreased in the CRS group, while increased in non-CRS individuals. Additionally, CRSwNP was correlated with a more prominent presence of *Staphylococcus aureus* and its EVs [81]. Research on bacteria-derived sEVs is a relatively new but growing field of knowledge that will contribute to a better understanding of the molecular basis of diseases and the search for new therapeutic solutions. Especially in inflammatory diseases of the respiratory tract, where the physiological microbiome meets environmental factors, including pathogenic microorganisms, this could be of importance.

#### 3.1.4. sEVs and Respiratory Viruses

Upper-airway viral infections are the most frequent illnesses of the respiratory tract, with adults experiencing the common cold 2–4 times a year and children 6–10 times a year [82]. Acute exacerbation of chronic airway conditions, especially asthma and COPD, are also frequent clinical problems. In both instances, viral infections are the most common cause [83]. On a cellular level, viruses can exploit the host’s machinery for vesicle biogenesis and use it to their advantage [84]; sEVs from infected cells carry viral genetic material, proteins, and, in some cases, whole viruses. As one vesicle contains several virus particles, this type of transmission is highly infectious [85]. For non-enveloped viruses, the phospholipid bilayer of the sEV serves as a protective barrier from neutralizing antibodies. To spread, viruses also exploit the cellular sEV release mechanism. This process prevents cytolysis, which is a very immunogenic event [8]. However, sEVs have a complex and contextual role in disease, as they can both mitigate and exacerbate the course of the disease. To that end, sEVs present in tracheobronchial mucus contribute to its antimicrobial function by presenting α-2,6-linked sialic acid on their surface, which is known to bind and neutralize human influenza virus particles [86].

Consistent with their role as messengers at the intersection of inflammation and immune response, sEVs released from cells during respiratory syncytial virus (RSV) infection were found to contain both viral RNA (mRNA, rRNA, and short non-coding RNA) and proteins. These sEVs were not infective and had no diagnostic value, but were able to induce chemokine release from monocytes and epithelial cells in vitro [87]. Similarly, human rhinovirus (RV) triggers the release of the pro-inflammatory protein Tensacin-C, which is associated with sEVs. This in turn leads to increased cytokine production by macrophages [88].

Paracrine communication between alveolar macrophages and lung epithelial cells plays an important role in the damage and repair of alveoli. Scheller et al. analyzed sEV-associated miRNAs in BALF from patients with influenza A induced acute respiratory distress syndrome (ARDS). In comparison to samples from healthy volunteers, four miRNAs were significantly deregulated. Most striking was the overexpression of miR17-5p, which was shown to enhance viral replication in vitro by downregulation of Mx1 antiviral factor [89]. In contrast, a beneficial role of sEV-associated miRNAs was described in a mouse model of LPS-induced acute lung injury. An intratracheal administration of sEVs that contained miRNA-126 derived from endothelial progenitor cells facilitated the regeneration of the alveolar epithelium [50]. Lastly, miRNAs associated with serum-derived sEVs were proposed as biomarkers of adenoviral pneumonia in the pediatric population [51].

### 3.2. Lower Airways

#### sEVs in Bronchial and Lung Diseases

EVs, including sEVs, participate in the regulation of immune cell functions during inflammatory diseases of the airways. Current research suggests that sEVs are important players in the pathogenic states of bronchial epithelium, such as the development of asthma; sEVs are released from airway epithelium and carry miRNAs (miR-34a, miR-92b, miR-210) that may impact the Th2-dependent immune response in asthma [52], and sEVs derived from mesenchymal stroma cells influence Treg suppression through the activation of peripheral blood mononuclear cells (PBMCs) to secrete IL-10 and TGF-β. Due to their capacity in the interaction with B cells and monocytes, but not with CD4+ T cells, sEVs may lead to Treg suppression. That might be of high importance in the asthma pathogenesis and in consequence be a potential therapeutic target [90]. In patients with asthma, dendritic cells treated with thymic stromal lymphopoietin (TSLP) secrete sEVs containing OX40 ligand. This triggers CD4+ T cell proliferation, increases the levels of IL-4, and directs the Th2 response [53]. Interestingly, it was shown that sEVs are also able to transfer mitochondrial components from myeloid-derived regulatory cells to T cells [91]. Other studies demonstrated the potential of EVs to transport mtDNA [92], as well as functional respiratory complexes [93]. In mouse models and cell culture models of allergy and asthma, increased levels of sEVs were demonstrated. Stimulation with epithelial-derived sEVs that carried IL-13 induced proliferation of inflammatory cells. Inhibition of sEV secretion also resulted in a decrease in inflammation [94].

Previous studies demonstrated that sEVs transfer proteins and miRNA between primary human tracheobronchial cells to the adenocarcinoma cells of the lung, contributing to the activity of recipient cells [95]. It was demonstrated that pro-inflammatory properties of BALF-derived sEVs may induce and foster the progress of inflammation in sarcoidosis. They carry increased levels of NRG1, which can be important for cell survival and proliferation, and are common in cancer [15]; sEVs from BALF of asthmatic patients carry an altered lipid composition, which may be important for the developed inflammation [54]. Other studies emphasize the role of sEVs in the acute lung injury (ALI) and ARDS generated in mouse models through inflammatory and non-inflammatory agents. Levels of EVs in BALF were elevated, affecting macrophages and enhancing lung inflammation [96]. In mice, macrophages that are involved in ALI pathogenesis secrete sEVs that stimulate other macrophages to produce TNF-α. In addition, lung epithelium-derived IL-25 downregulates proteins involved in sEV secretion in macrophages, and consequently inhibits sEV release and TNF-α secretion [97]. Analysis of BALF-derived sEVs from asthmatic and healthy patients indicated changes in their properties. In the context of asthma, sEVs contribute to an upregulation of cytokines and leukotrienes in airway epithelium [55].

Studies conducted on models of asthma and COPD have shown that bacterial and viral infections of the respiratory system lead to an increased release of EVs and a heightened secretion of cytokines, which stimulate neutrophils [98]; sEV secretion from airway epithelia is subject to regulation. Cigarette smoke can upregulate this process. In contrast, thiol antioxidants may inhibit this process and have a beneficial effect in COPD and other respiratory pathologies [99]. Another study examining plasma-derived EVs, including sEVs, isolated from patients with COPD, tobacco users, and non-smoking healthy individuals showed differential miRNA expression, which suggests their use as biomarkers [56].

## 4. Future Perspectives: Diagnostic and Therapeutic Potential

sEV research is a relatively new but rapidly growing field of research, and sEVs emerge as promising prognostic, diagnostic, and therapeutic tools for future clinical use [100,101]. Despite the great potential of using sEV-based methods in clinical practice, to the best of our knowledge, none have been approved so far. In inflammatory airway diseases, sEVs isolated from different body fluids, such as serum, plasma, nasal lavage fluid, or bronchoalveolar lavage fluid, have been shown to carry a complex molecular cargo that has great potential to be utilized for diagnostic purposes. One example are the specific miRNAs that are found in sEVs isolated from the serum of children suffering from human adenovirus-induced pneumonia [51]. Another example for the prognostic and diagnostic value of sEVs are the airway-associated cancers. Pleural lavage-derived EVs carry a signature of miRNAs (miRNA-1-3p, miRNA-144-5p, miRNA-150-5p) that are promising biomarkers for lung cancer diagnosis [102]. Serum-derived sEVs in non-small lung cancer carry miR-1269a, which serves as a diagnostic marker, and also plays an oncogenic role by regulating *FOXO1* [103]. A significant research effort will be necessary to unravel the complexity of biological functions of sEVs. Safe usage in clinical applications will require a standardization of isolation procedures from clinical material and cell culture, as well as unification of analytic methods.

## 5. Summary

A growing body of evidence suggests that sEVs might be useful as biomarkers in many diseases [102,104,105,106], as well as prognostic agents providing information about the phase of disease or predicted therapy outcomes [107,108,109]; sEVs transfer specific cargo components depending on the cell of origin, and ensure a safe environment for transported compounds. This allows their delivery to both neighboring and distant cells, which is an important modality of communication between tissues and organs. Furthermore, sEVs are involved in antigen presentation, and can activate immune cells, which allows them to stimulate or inhibit immunological pathways; sEVs are considered safe, are well-tolerated by the organism, and, therefore, show great potential for drug transport or vaccination. Regulation of sEV secretion that enhances or inhibits their release might be an interesting therapeutic strategy. Finally, their ubiquitous presence in body fluids gives hope for their use in liquid biopsy [33].

## Figures and Tables

**Table 1:** Comparison of articles from our literature search with emphasis on the source of sEVs, their cargo, and biological effects. Abbreviations: CRSwNP: chronic rhinosinusitis with nasal polyps; CRSsNP: chronic rhinosinusitis without nasal polyps; P-gp: permeability glycoprotein; TLR: toll-like receptor; PAPP-A: pappalysin A; LncGAS5: long-noncoding RNA GAS5); NLF: nasal lavage fluid; ADAM10: disintegrin and metalloprotease 10; MEE: middle ear effusion; MUC5B: mucin 5B; hNECS: human nasal epithelial cells; PPAR: peroxisome proliferator-activated receptor; SERPINE1: serpin family E member 1; PERP: P53 apoptosis effector related to PMP22; PLTP: phospholipid transfer protein; HMEEC: human middle-ear epithelial cells; hRNP: heterogenous nuclear ribonucleoprotein; miR/miRNA; microRNA; RANKL: receptor activator of nuclear factor—kappa B ligand; hCPFS: human cholestatoma perimatrix fibroblasts; ARDS: acute respiratory distress syndrome; EPC: endothelial progenitor cells; MPO: myeloperoxidase; NHBE: normal human bronchial epithelial cells; BALF: bronchoalveolar lavage fluid; NRG1: neuregulin 1; COPD: chronic obstructive pulmonary disease; tRNA: transfer RNA; piRNA: Piwi-interacting RNA; snRNA: small nuclear RNA; snoRNA: small nucleolar RNA.

Reference	Disease; Source of sEVs	sEVs Cargo	Possible Biological Function
Nocera et al. [34]	CRSwNP; nasal mucus	P-gp	Possible regulation Th2 cytokine production
Mueller et al. [39]	CRSwNP; nasal mucus	Cystatin-SN, Peroxiredoxin-5, Glycoprotein VI	Cysteine protease inhibition Innate immune regulation Activation of TLRs Antioxidant activity Activation of platelets
Miyake et al. [40]	CRSwNP; CRSsNP nasal mucus	Cystatin-1 Cystatin-2	Epithelial barrier functions
Mueller et al. [41]	CRSwNP; nasal mucus	PAPP-A	Epithelial proliferation Polyp growth
Mueller et al. [42]	CRSwNP; nasal mucus	Serpins	Polyp fibirin deposition
Zhu et al. [43]	Allergic rhinitis; nasal mucus, nasal epithelial cells	LncGAS5	Suppression of CD4+ to Th1 differentiation, promoted Th2 differentiation
Zhang et al. [44]	CRSwNP;NLF	ADAM10	Angiogenesis Vascular permeability
Val et al. [45]	Otitis media, MEE	miR-233 MUC5B	IL- 8 activity Neutrophil functions Innate immune responses
Zhou et. al. [46]	CRSwNP, CRSwNP + asthma; hNECs	Proteins involved in p53 and PPAR signaling pathways SERPINE1, PERP, PLTP, ladinin-1, myosin-9	Tissue repair and remodeling Immune system signaling Immune responses to viruses and bacteria Cell cycle signaling Cell growth and replication Cell cycle control
Val et al. [47]	Haemophilus influenzae infection; HMEEC	hnRNP A2B1 hnRNP Q miR-378-a-3p miR-378i miR-200a-3p miR-378g miR30d-5p miR-222-3p	Immunity regulation Inflammatory pathways Angiogenesis Neutrophil adhesion
Gong et al. [48]	Middle ear cholestatoma; keratinocites	miRNA-17	Upregulation of RANKL Osteoclast differentiation
Li et al. [49]	Cholestatoma; hCPFs	miR-106b-5p	Angiogenesis Overexpression of Angiopoietin-2 in human umbilical vein endothelial cells Tube formation Cell migration
Zhou et al. [50]	ARDS; EPC	miR-126	Reduction of permeability and inflammation Reduced MPO activity Lung injury protection
Huang et al. [51]	Pneumonia; Adenovirus Infection; serum	miR-450a-5p/miR-103a-3p, miR-103b/miR-98-5p	Immunoregulatory function
Bartel et al. [52]	Asthma; NHBE nasal lavage	miR-34a, miR-92b, miR-210	Th2 response Dendritic cell activity
Huang et al. [53]	Asthma; Dendritic cells	OXO40 ligand	CD4+ T cell proliferation Increase IL-4 Th2 response
Quazi et al. [15]	Sarcoidosis; BALF	NRG1	Inflammation Proliferation Cell survival
Hough et al. [54]	Asthma; BALF	lipids	Inflammation
Torregrosa et al. [55]	Asthma; BALF	enzymes for leukotriene biosynthesis	Upregulation of cytokines and leukotrienes in airway epithelium
Sundar et al. [56]	COPD; plasma	miRNAs tRNAs piRNAs snRNAs snoRNAa	Inflammation Extracellular matrix and tissue remodeling
